# Intact *Mycobacterium leprae* Isolated from Placenta of a Pregnant Woman, China

**DOI:** 10.3201/eid2608.191149

**Published:** 2020-08

**Authors:** Ajay Vir Singh, Harpreet Singh Pawar, Rajbala Yadav, Devendra Singh Chauhan

**Affiliations:** Indian Council of Medical Research–National JALMA Institute for Leprosy and Other Mycobacterial Diseases, Agra, India

**Keywords:** Bacteria, tuberculosis and other mycobacteria, leprosy, China, newborn, placenta, maternal and child health, Hansen disease, Mycobacterium leprae, transmission

**To the Editor:** Chen et al. (*1*) reported intact *Mycobacterium leprae* in homogenate of placenta of a pregnant woman with untreated histoid leproma, highlighting the effectiveness of the placental barrier in stopping vertical transmission of leprosy (Hansen disease). However, reports in the published literature indicate that this claim is not absolutely correct.

Several early studies provided evidence of transplacental transmission of *M. leprae*; these studies revealed *M. leprae* in umbilical cords (25/104) (*2*) and cord blood (10/12) (*3*) of neonates born to mothers with leprosy, as well as in the placentae (57/104 and 9/12) (*2**,**3*) of those mothers (*2*,*3*). Furthermore, transplacental infection with *M. leprae* has been supported by an increased concentration of IgA in cord blood (*4*) and *M. leprae* IgA and IgM in cord serum (*5*) of babies of mothers with leprosy. These observations indicate that in some mothers with leprosy, whole *M. leprae*, its antigens, or both can cross the placenta, possibly inducing the fetal immune system to produce antibodies against *M. leprae* antigens. Therefore, we believe that vertical transmission of *M. leprae* is a complex, uncommon, and multifactorial event that might depend on the presence of *M. leprae* in maternal blood, maternal and fetal immune responses, fetal gestational age at infection, and other placental factors.

Consequently, the claim of Chen et al. (*1*) needs to be read with attention to the limitations of the underlying data and might not be generalizable to all mothers with leprosy. Further studies are needed to clarify the mechanisms of transplacental transmission of leprosy. The follow-up care of newborns of mothers with leprosy is necessary for early detection of the disease and to ensure appropriate general healthcare, especially considering that babies of mothers with leprosy have lower fetoplacental weights, slower growth, more fatal infections, and higher rates of infant mortality than those of mothers without leprosy.
